# Determinants of Healthcare Providers’ Notification, Prevention and Perception Scores Under the Notifiable Disease Surveillance System (NDSS) in Jazan, Saudi Arabia

**DOI:** 10.3390/healthcare14131936

**Published:** 2026-07-01

**Authors:** Moatasem Fahd Hakmi, Alaa Mathkour, Yousef Zahrani, Rayan Ibrahim H. Binduhayyim, Saad S. Alqahtani, Muhammad Zahid Iqbal

**Affiliations:** 1 Public Health Administration, Jazan Specialist Hospital, Health Holding Co., Jazan Health Cluster, Jazan 82543, Saudi Arabia; moatasem.fahd@gmail.com (M.F.H.); amathkour@yahoo.com (A.M.); 2 Public Health Department, COAMS, King Khalid University, Abha 62529, Saudi Arabia; y.zahrani@kku.edu.sa; 3 Department of Dental Technology, COAMS, King Khalid University, Abha 62529, Saudi Arabia; rihasan@kku.edu.sa; 4 Department of Clinical Pharmacy, College of Pharmacy, King Khalid University, Abha 61421, Saudi Arabia

**Keywords:** notifiable disease surveillance system (NDSS), HESN + training, disease notification, healthcare providers, surveillance competency, public health surveillance, epidemiology training, case management, preventive practices, cross-sectional study, Saudi Arabia, health information systems

## Abstract

**Background:** Effective notifiable disease surveillance depends on healthcare providers’ ability to identify and report cases. Evidence on factors associated with surveillance performance remains limited in high-risk regions such as Jazan, Saudi Arabia. **Objective:** This study examined factors associated with healthcare providers’ notification and case management skills, prevention and training competencies, and perceptions toward the Notifiable Disease Surveillance System (NDSS). **Methods:** A cross-sectional analytical study was conducted among 420 healthcare providers working in government hospitals and primary healthcare centers in Jazan. Data were collected using an adapted self-administered questionnaire. Composite scores were analyzed using t-tests, ANOVA, and linear regression. **Results:** Higher NDSS-related scores were associated with professional category, HESN+ training, epidemiology or surveillance training, and years of experience. In multivariable analyses, HESN+ training, formal epidemiology or surveillance training, professional category, and experience remained associated with notification and prevention-related outcomes. Notification and case management skills were also associated with more favorable NDSS perceptions. **Conclusions:** NDSS-related competencies and perceptions were associated mainly with training exposure, professional role, and experience. Strengthening HESN+ training and integrating surveillance education into workforce development programs may support disease reporting and surveillance capacity in Jazan.

## 1. Introduction

An active decision support mechanism constitutes public health surveillance [1]. Instead of gathering reports and data, this approach gathers data to improve health outcomes and disease-related decisions for whole populations [2]. When disease monitoring systems work well, they help detect early outbreaks of diseases, track how diseases are spreading, and enable healthcare providers to take quick actions for further prevention [3,4]. The Notifiable Disease Surveillance System (NDSS) is a public health reporting system designed to facilitate the timely detection, notification, monitoring, and control of communicable diseases of public health importance. As it focuses on urgent public health actions for illnesses that require immediate reporting, the Notifiable Disease Surveillance System has particular importance within this framework [5]. Due to the sense of urgency or emergency it carries, a notice-based disease reporting system is different from other reporting systems [6].

Simply setting up a monitoring system, which is what we call a surveillance system, does not mean that it gives optimal performance. Regardless of having laws and electronic devices, optimization of the system depends on the persons who detect the illnesses and trigger the notifications [6,7]. This crucial position is held by healthcare professionals. Legal authority has been given to healthcare providers to detect notifiable diseases and inform the healthcare authorities regarding this [8,9]. Effective surveillance happens when healthcare providers use their clinical reasoning, understand how to report the data, and feel certain about how to use those established systems for reporting. These human elements are less visible than infrastructure, but arguably more decisive [10].

Many health systems still miss some cases and face underreporting of notifiable diseases, which makes the systems less effective [11]. Notifications of reporting may not be on time, surveillance data may be incomplete, and reporting quality may differ from other regions and states [12]. Inconsistent practices diminish surveillance sensitivity and delay outbreak recognition. Moreover, evidence has shown that underreporting is not only due to a lack of knowledge [13]. Instead, reporting behavior is influenced by multiple factors, due to individual competencies, institutional limits, and professional expectations [14]. In many contexts, reporting systems have improved reporting efficiency, but in some cases, they have failed to deliver the expected outcomes [15].

Over time, interest has turned to explaining variations in compliance among healthcare providers, and professional training and role have consistently been identified as important contributors. Performance variance has also been associated with years of experience, exposure to epidemiological training, workload pressures, and familiarity with surveillance systems [16,17]. In many settings, physicians and public health practitioners show greater regularity in reporting than their counterparts in nursing and laboratory roles [11,16]. Although the trend is well-documented, it is still up for debate whether this discrepancy is due to training differences, role expectations, or institutional culture. Epidemiology training is frequently connected with higher confidence levels and faster notification performance [17]. Based on these data, it is possible that workforce characteristics could have impacts equal to those of policy frameworks.

Following challenges posed by emerging and re-emerging infections, Saudi Arabia has prioritized and expanded funding for communicable disease surveillance systems [18,19,20]. The establishment of the electronic health surveillance network aims to rationalize the reporting mechanism; it makes reporting faster and reduces delays and it helps coordination between institutions and health authorities [21]. Even though the systems have all the technical abilities required to report or document the data, users are not fully taking advantage of these capabilities [21]. Studies have shown that the electronic system is not being used equally everywhere; training to use the systems has not been sufficient and organizational barriers make it hard to fit the system into the daily routine of clinical work [16]. As primary healthcare systems are very busy and do not have much technical support, they may find it hard to use and incorporate electronic systems in their routine practice [22].

Jazan has shown evidence of higher rates of infectious and vector-borne diseases, which creates additional challenges and considerations for disease surveillance and control over this region [23]. The high disease rates are linked to climate changes and movements of humans across the borders, which can cause the increased spread of disease [24]. In such an environment, where diseases spread easily, reporting delays can lead to outbreaks growing faster, delayed control measures and increased numbers of people suffering from infections [16]. Even though there are multiple health challenges existing in Jazan, there is still limited research data available on what affects the performance of the Notifiable Disease Surveillance System. By performing descriptive research, researchers have been able to understand what people know and how they feel or think about the surveillance system [25,26]. Recent advances in healthcare systems research have highlighted the growing importance of digital technologies, workforce capacity development, and data-driven approaches for improving healthcare delivery and public health outcomes. As health systems increasingly rely on digital platforms and integrated information systems, understanding the factors that influence healthcare providers’ engagement with disease surveillance systems has become increasingly important [27,28,29]. The NDSS supports the timely detection, reporting, and monitoring of communicable diseases, enabling public health authorities to respond rapidly to outbreaks and other public health threats. On the other hand, very few studies have explored the factors that can affect how people report cases, practice prevention or think about surveillance systems.

Therefore, a more focused research effort regarding factors influencing outcomes should be done. If the performance of a surveillance system across regions varies, then we should examine the cause rather than making assumptions about it. Previous studies conducted in Saudi Arabia and other countries have largely focused on descriptive evaluations of healthcare providers’ knowledge, awareness, compliance, or perceptions regarding communicable disease surveillance systems. While these studies have identified gaps in reporting practices and training exposure, only a limited amount of research has analytically examined the determinants associated with NDSS-related competencies and perceptions across multiple professional groups, particularly in high-risk regions such as Jazan. In addition, few studies have simultaneously explored notification performance, preventive competencies, and surveillance perceptions within a unified analytical framework.

Accordingly, the present study extends the existing literature by examining the socio-demographic, professional, and training-related factors associated with multiple dimensions of NDSS engagement among healthcare providers in Jazan, Saudi Arabia. This research aimed to identify socio-demographic factors, professional attributes, and variables related to training in the use of surveillance systems, and their association with notification performance, case management ability, and preventive and training skills, in addition to determining the perceptions of the NDSS system across government hospitals and primary healthcare facilities in Jazan, Saudia Arabia.

By emphasizing predictors rather than descriptive levels alone, evidence may be generated that informs targeted capacity-building and surveillance-strengthening strategies in settings characterized by elevated communicable disease risk.

## 2. Methodology

### 2.1. Study Design

A cross-sectional analytical design was employed in this study. Notification skills, preventive competencies, and perceptions toward the Notifiable Disease Surveillance System were assessed simultaneously within a defined population of healthcare providers. Data were collected at a single point in time, allowing associations between outcome measures and selected socio-demographic and professional factors to be examined. While causal relationships could not be inferred, patterns of association were identified and evaluated.

### 2.2. Study Setting

Researchers collected data from government health facilities across the Jazan region in southwestern Saudi Arabia. The Ministry of Health oversees healthcare delivery in this area through a linked network of hospitals and primary healthcare centers that provide both preventive services and clinical care. According to official Ministry records the region includes 21 public hospitals and 165 primary healthcare centers. These facilities serve as the main locations for identifying suspected cases managing patients and submitting formal notifications of notifiable diseases to public health authorities.

### 2.3. Study Population

Healthcare professionals working in government hospitals and primary healthcare centers across the Jazan region formed the study population. Physicians, nurses, public health workers and laboratory staff were included when their duties involved identifying, managing, or reporting communicable and notifiable diseases either directly or through routine support roles. Those who were on long-term leave during the data collection period and those who chose not to participate were excluded from the sample.

### 2.4. Sample Size Determination

The estimation of the required sample size was based on standard parameters of 95 percent confidence and 5 percent allowable error. Without existing regional estimates, a 50 percent prevalence was assumed to produce the maximum sample size. Calculation was performed using the Raosoft online sample-size calculator (Raosoft Inc., Seattle, WA, USA, Available online: http://raosoftcalculator.com/ (accessed on 23 June 2026)). These criteria suggested that there should be at least 384 participants. This numeric goal was increased to accommodate possible non-response and incomplete submissions. In the end, the survey comprised 420 healthcare providers in all.

### 2.5. Sampling Technique

To recruit participants, a non-probability convenience sampling strategy was employed. Participants were selected based on availability rather than randomly selected, so the sample may not fully represent all healthcare workers. However, the study still included different professions and types of facilities, which adds depth to the findings.

### 2.6. Data Collection Instrument

The researchers collected data using a standardized survey that participants filled out themselves. The survey was adapted from similar questionnaires used in earlier studies in Saudi Arabia and South Africa [11,16]. The researchers adapted the questionnaire so that it would be suitable for the local setting and context. The tool was carefully designed and organized to gather information about healthcare workers’ skills and their views about the disease surveillance system. In Saudi Arabia, disease surveillance activities are supported by the Health Electronic Surveillance Network (HESN+), a national electronic platform used for reporting, monitoring, and managing notifiable disease data across healthcare facilities and public health authorities. Modifications were made to ensure alignment with the local healthcare context, particularly the structure and functionality of the Health Electronic Surveillance Network (HESN+). Wording adjustments were introduced to improve clarity and contextual relevance.

The questionnaire was composed of multiple topics, including perceptions of the surveillance system, preventative and training competencies, notification and case management procedures, and sociodemographic and professional characteristics. The questionnaire was designed to assess both what healthcare workers know about reporting diseases and how they actually use the electronic surveillance system in practice. The researchers combined several related survey questions to create overall scores that measured how well participants report cases, how capable they are in matters of prevention, and how they perceive the surveillance system.

Participants were recruited through direct distribution of the survey link to healthcare providers working in selected government hospitals and primary healthcare centers. The questionnaire was disseminated via institutional communication channels, including email and WhatsApp groups, to maximize reach across facilities. Due to the open distribution approach, it was not possible to determine the exact number of individuals who received the survey, and therefore a response rate could not be calculated. The questionnaire used in this study is provided as Supplementary File S1.

### 2.7. Validity and Reliability

The questionnaire covered the topic thoroughly and appropriately, and this quality was improved or reinforced because it was adapted from tools that had already been tested and validated in previous studies [11,16]. The questionnaire was carefully scrutinized to make sure all questions matched the study’s goals and were relevant to the local surveillance system.

We made small revisions to make sure the questionnaire was clear and appropriate for the local context. Prior to the main data collection, a pilot test was conducted among a small group of healthcare professionals to assess the clarity, relevance, and comprehensibility of the questionnaire items. Feedback obtained from the pilot phase was used to refine the instrument.

Reliability regarding internal consistency was assessed using Cronbach’s alpha coefficient for each domain. The values were as follows: notification and case management domain (α = 0.87), prevention and training domain (α = 0.82), and perception domain (α = 0.79); these indicate acceptable-to-good reliability across all domains. All parts of the questionnaire were statistically reliable, meaning the questions within each section worked well together and consistently measured what they were supposed to measure.

Although the questionnaire demonstrated acceptable internal consistency reliability, additional psychometric validation procedures such as exploratory or confirmatory factor analysis and criterion-related validation were not performed. Therefore, the measured domains may partly reflect self-perceived competency, confidence, and attitudes toward the NDSS rather than objectively verified performance measures. Future studies are encouraged to conduct more comprehensive psychometric evaluations of the instrument.

### 2.8. Data Collection Procedure

**Figure 1:**
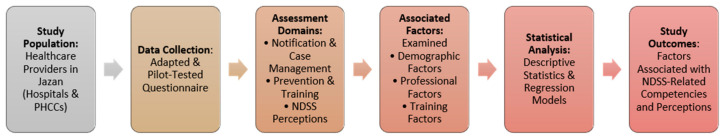
Overview of the study’s workflow.

Google Forms surveys were generated to facilitate data collection, and the questionnaires were circulated among participants. Healthcare professionals who were qualified for the study received a link to questionnaire through email or via WhatsApp. Questionnaire links were distributed in government hospitals and in the primary healthcare centers of Jazan. Surveys started with the introduction of the study and then mentioned the voluntarily nature of participation. The information-handling processes of the study ensured protection of the participants’ privacy and anonymity. The completion and submission of the survey by the participants indicated their consent to be included in the study. Responses were collected during a fixed time-frame and participation was monitored, and then the responses were compiled for analysis. An overview of the study’s workflow is presented in Figure 1.

### 2.9. Outcome Measures

The study’s main focus was on three overall scores. These scores were created by combining answers from selected questionnaire questions; these combined scores were the main variables analyzed in the present study. Questions that measured the same idea were grouped into sections to construct the domain representing a specific aspect or category.

The total score for each domain showed participants’ skills relating to or perception of that specific area. The scoring system was designed in such way that a high score represented stronger skills, better performance, and a more positive perception. When analyzing the data, the researchers treated the overall scores as numerical variables that vary along a scale, allowing them to use more advanced statistical tests. Each outcome domain was constructed by aggregating responses from multiple questionnaire items representing the same conceptual construct. The notification and case management domain consisted of 12 items, the prevention and training domain included 7 items, and the perception domain comprised 10 items.

Responses were scored using a structured scale, and item scores were summed to generate composite domain scores. No weighting was applied, and all items contributed equally to their respective domain scores.

The resulting composite scores were treated as continuous variables in the analysis. This approach was considered appropriate due to its aggregation of multiple items, which approximates a continuous distribution and is consistent with practices in similar studies.

### 2.10. Data Management and Statistical Analysis

The researchers downloaded the responses from Google Forms and entered them into the Statistical Package for the Social Sciences, version 28 for analysis. Before running any statistical tests, they reviewed the dataset to check the accuracy, completeness and logical consistency of the information. Missing values were reviewed and corrected where possible to prepare the data for further analysis.

Basic descriptive statistics were then used to summarize the characteristics of the study participants. Measures associated with continuous data were presented as means with corresponding standard deviations, while categorical data were described using frequencies and percentages. Differences in mean outcome scores between two groups were examined using independent sample t tests, whereas comparisons across more than two groups were conducted using one-way analysis of variance.

To identify determinants of notification and prevention and perception outcomes, linear regression modeling was applied. Variables with conceptual or theoretical relevance were included in the regression analyses. Statistical significance was established by demonstrating a two-tailed probability value below 0.05.

### 2.11. Ethical Considerations

Ethical approval for this study was obtained from the Research Ethics Committee of King Khalid University (HAPO-06-B-001), Saudi Arabia (Approval No. ECM#2024-31108). Participation was voluntary, and informed consent was obtained from all participants prior to data collection. Confidentiality and anonymity of participant information were maintained throughout the study.

### 2.12. Statistical Analysis

Data analysis was conducted with the IBM Statistical Package for Social Sciences, version 28 (Armonk, New York, NY, USA). Prior to conducting parametric tests, assumptions of normality and homogeneity of variance were assessed through visual inspection and statistical indicators. The distributions of composite scores were found to be approximately normal. Multicollinearity among independent variables in regression models was evaluated using variance inflation factors (VIF), and no significant multicollinearity was observed. Model diagnostics were performed through examination of residuals to ensure appropriate model fit. Variables included in the multivariable regression models were selected based on theoretical relevance and statistical significance in univariate analyses. Linear regression analyses were performed using unstandardized regression coefficients (B) with corresponding 95% confidence intervals to estimate associations between explanatory variables and NDSS-related outcome scores. Participant characteristics were summarized through the application of descriptive statistical methods. Categorical data were reported in terms of frequencies and percentages, whereas continuous variables were described using means and standard deviations.

Independent sample t-tests were used to compare mean NDSS scores between two groups, and one-way analysis of variance (ANOVA) was applied for comparisons across more than two groups. Independent associations between explanatory variables and the notification, prevention, and perception scores were evaluated using linear regression analysis. Statistical significance was defined by a two tailed *p* value below 0.05.

## 3. Results

A total of 420 healthcare providers participated in the study. Participants represented different professional groups, including physicians, nurses, laboratory personnel, and public health professionals, all working in government hospitals and primary healthcare centers across the Jazan region. The sample included individuals with varying levels of professional experience and surveillance-related training and different educational backgrounds. Detailed demographic and professional characteristics of the study participants are presented in Table 1.

### 3.1. Factors Associated with Notification and Case Management Skills

The associations between socio-demographic and professional characteristics and notification and case management skills scores are presented in Table 1. Statistically significant associations were observed between notification and case management skills and professional category, years of experience, training on the Health Electronic Surveillance Network (HESN+), and having a formal educational degree or formal training in epidemiology or disease surveillance. Physicians and public health professionals demonstrated higher mean notification and case management scores compared to nurses and laboratory staff. Healthcare providers who had received training on HESN+ showed significantly higher mean scores than those who had not received such training. In addition, participants with formal epidemiology or surveillance training achieved higher scores compared to those without such training. Years of experience showed a positive association with notification and case management scores, while gender was not significantly associated.

### 3.2. Factors Associated with Prevention and Training Skills

The associations between socio-demographic and professional characteristics and prevention and training skills scores are shown in Table 2. Statistically significant differences in prevention and training scores were observed according to professional category, training on HESN+, and having formal education or training in epidemiology or disease surveillance. Public health professionals achieved the highest mean prevention and training scores among all professional groups. Healthcare providers who had received HESN+ training demonstrated higher mean prevention and training scores compared to those who had not received training. Participants with formal epidemiology or disease surveillance training also showed higher scores. No statistically significant association was observed between prevention and training scores and gender.

### 3.3. Factors Associated with Perceptions Toward the NDSS

The associations between socio-demographic and professional characteristics and perception scores relating to the NDSS are presented in Table 3. Significant associations were observed between perception scores and professional category, training on HESN+, and having formal education or training in epidemiology or disease surveillance. Healthcare providers who received HESN+ training reported more positive perceptions toward the NDSS compared to those who did not receive training. Public health professionals demonstrated higher perception scores than other professional groups. Years of experience showed a positive trend with perception scores, while gender was not significantly associated.

### 3.4. Predictors of Notification and Case Management Skills

The results of the linear regression analysis identifying predictors of notification and case management skills are presented in Table 4. In the multivariable model, professional category, training on HESN+, formal education or training in epidemiology or disease surveillance, and years of experience were identified as significant predictors of notification and case management skills. Healthcare providers who had received HESN+ training were more likely to have higher notification and case management scores, after adjusting for other variables.

### 3.5. Predictors of Prevention and Training Skills

The linear regression analysis for predictors of prevention and training skills is shown in Table 5. Professional category, training on HESN+, and formal education or training in epidemiology or disease surveillance were significant predictors of prevention and training skills. Participants who received training demonstrated higher prevention and training scores compared to those without training.

### 3.6. Predictors of Perception Toward NDSS

The predictors of perception toward the NDSS identified through linear regression analysis are presented in Table 6. Training on HESN+ and professional category were significant predictors of perception scores. Healthcare providers who had received HESN+ training demonstrated more positive perceptions toward the NDSS compared to those who had not received training.

## 4. Discussion

An analytical perspective was adopted in this study aiming to explore determinants associated with performance within the Notifiable Disease Surveillance System. Finding factors that seem to influence notification and preventive and perception outcomes was prioritized over focusing only on descriptive knowledge levels or reported practice. The findings show clear differences in the capabilities of different healthcare workers when it comes to the Notifiable Disease Surveillance System (NDSS). The differences in NDSS-related capabilities were measurable and were associated with training exposure, years of experience, and professional role.

Professional category was proven to be a reliable predictor across all outcome domains. Doctors and public health professionals scored better, as compared to nurses and laboratory staff. Similar findings were seen in a study conducted in Saudia Arabia, where doctors were consistently more aware of reporting rules, compared to other healthcare professionals, because the doctors receive more training in surveillance and case definitions [16,25]. Research from other countries has indicated that healthcare workers who officially have public health responsibilities are more likely to actively take part in disease surveillance [11]. Even though nurses and laboratory staff play key roles in patient care and identifying disease, they are often seen as not being trained in disease surveillance. Similarly, these discrepancies have been noted in previous studies, where reporting systems give more responsibility or importance to certain professional groups, which could create imbalance in participation and performance [13,16].

Training on the Health Electronic Surveillance Network (HESN+) was one of the strongest factors associated with higher reporting skills, preventive measures, and more favorable surveillance system-related outcomes. Healthcare workers who received training performed better in reporting and prevention tasks; they also had a more positive attitude towards the surveillance system. Similar Saudi studies report that insufficient training regarding the electronic system causes hindrance in use, especially in primary health care settings [13,16,21]. Similar findings were seen in other studies where it was reported that alone, technology is insufficient; improvements in reporting and speed emerged only when structured training programs were introduced [26]. These findings suggest that the presence of a surveillance system alone may not necessarily be associated with improved performance, without adequate training and ongoing support for healthcare workers.

Healthcare professionals who had formal education or training in epidemiology or disease surveillance tended to perform better and have stronger NDSS-related outcomes. In addition to reporting more favorable opinions of the system, participants who received such training scored higher in the notification and preventative domains. Similar trends were seen in previous studies where the results have consistently demonstrated that early exposure to public health concepts deepens appreciation of surveillance functions and encourages more consistent reporting behavior [3,17]. Another similar study in other contexts has also shown that inadequate epidemiological training leads to underreporting and poorer system performance [11]. Evidence from the literature suggests that when surveillance education is incorporated into sustained professional development efforts, healthcare providers demonstrate higher levels of involvement and greater consistency in reporting activities regarding notifiable disease surveillance [26].

Field experience had a positive association with the management of cases and reporting. This positive link or association may reflect improved procedural awareness, which earlier investigations have identified as an important influence [16]. Even so, having years of experience does not guarantee optimal performance, without ongoing support. Even when experience and other factors were taken into account, training continued to be a powerful predictor of better performance. This means training has its own independent effect. In those contexts, where staff members continuously move around or leave, relying only on experience-based learning is not stable or reliable.

Both exposure to training and professional role have impacts on perceptions of the NDSS. Individuals who underwent HESN-specific instruction or who had formal epidemiological training tended to report more supportive views of the surveillance system. Because active participation is linked to perceived usefulness and manageability, prior research has connected favorable opinions about surveillance systems with better reporting behavior [11,26]. Complicated systems, lack of feedback, and high workload have been connected to reduced reporting [13,14]. According to the current research, training exposure was associated with greater technical proficiency and more favorable attitudes toward reporting compliance. Multivariable modeling demonstrated that, despite adjustment for possible confounding effects, professional category and training exposure persisted as significant explanatory factors. This supports the idea that focused workforce development initiatives have a higher chance of improving surveillance performance than do regulatory mandates alone. Consistent findings in earlier surveillance research have underscored the pivotal role of workforce capacity-building in ensuring system effectiveness [10,26].

While statistical significance was observed across several variables, the magnitudes of the associations provide additional insight into their practical importance. Training-related variables, particularly HESN+ training, demonstrated consistently stronger effects compared to other factors, suggesting that targeted training interventions may yield meaningful improvements in surveillance performance.

Some variables that were significant in univariate analysis lost significance in multivariable models. This may be explained by confounding and shared variance among predictors, particularly between professional category, training exposure, and years of experience.

All things considered, the results are consistent with national and international research showing that professional positioning, individual competency, and institutional support systems influence NDSS success [25]. In addition, the study brings forward evidence from Jazan, a setting distinguished by increased vulnerability to communicable diseases [23,24]. The observed and consistent relationship between training and NDSS-related performance indicates a feasible avenue for programmatic action. Enhancing HESN training coverage, incorporating surveillance competencies into continuous professional development frameworks, and reducing disparities among nursing and laboratory staff may improve the accuracy and promptness of regional disease notification.

The findings of this study align with broader healthcare system research emphasizing the importance of workforce preparedness and effective utilization of digital health infrastructure. As healthcare systems continue to adopt technology-enabled approaches for improving service delivery and public health performance, strengthening surveillance-related competencies among healthcare providers remains a critical component of health system resilience [27,28,29].

The observed association between HESN+ training and higher NDSS-related competency scores has important implications for healthcare policy and workforce development in Saudi Arabia. As the Kingdom continues to strengthen its public health infrastructure and digital health systems, ensuring that healthcare providers are adequately trained in electronic disease surveillance should remain a strategic priority. Structured HESN+ training programs may contribute to more standardized reporting practices, improved familiarity with surveillance procedures, and greater confidence in the use of electronic notification systems. Incorporating surveillance competencies into continuing professional development programs and routine workforce training may help strengthen surveillance capacity across healthcare settings. These efforts may be particularly important in high-risk regions such as Jazan, where timely detection and reporting of communicable diseases are essential for effective public health response.

The findings should also be interpreted in light of potential biases. Self-reported data may be subject to recall bias and social desirability bias, potentially leading to overestimation of competencies. Additionally, differences observed between professional groups may reflect institutional role expectations and access to training rather than intrinsic differences in capability.

Several strengths of this study should be acknowledged. However, certain limitations should also be considered. First, causal inference is not possible due to the cross-sectional design, and associations cannot be interpreted as causal relationships. Second, the use of self-reported data may introduce recall bias and social desirability bias, potentially influencing the accuracy of responses. Third, the non-random convenience sampling approach introduces the possibility of selection bias, as participation may have been more likely among individuals who were more motivated, more familiar with digital tools, or more engaged in surveillance-related activities. Consequently, the findings may not fully represent all healthcare providers in the region, limiting the generalizability of the results to broader healthcare settings. Another limitation relates to the psychometric evaluation of the adapted questionnaire. While the instrument was adapted from previously used surveys, pilot tested, and demonstrated acceptable internal consistency, advanced psychometric validation methods such as factor analysis and criterion validation were not conducted. Consequently, some domains may reflect self-reported perceptions and confidence rather than objectively measured competency. Future research should further validate the instrument using comprehensive psychometric approaches.

## 5. Conclusions

The findings suggest that engagement with the Notifiable Disease Surveillance System in the Jazan region is determined by multiple interconnected factors, not by any single influence alone. Differences in professional role, prior HESN training, academic background in epidemiology, and years of work experience were all linked to variations in reporting practice, case management, prevention capacity, and general views about the NDSS. Among these factors, structured training in electronic surveillance demonstrated one of the strongest associations with NDSS-related outcomes and may represent an important target for workforce development strategies.

The findings highlight the importance of strengthening workforce capacity through structured surveillance education and continued professional development initiatives. In regions with elevated communicable disease risks, such efforts may support more effective disease reporting and contribute to stronger public health surveillance systems.

## Figures and Tables

**Table 1:** Relation between demographic data and scores for notification/case management skills on the NDSS.

Item	Notification and Case Management Skills Score	*p*-Value
**Professional category**		
Physician	46.72 ± 13.54 ^a^	**<0.001**
Nurse	37.66 ± 13.69 ^b^	
Public health specialties	50.56 ± 11.76 ^a^	
Medical laboratory	38.86 ± 15.55 ^b^	
**Gender**		
Male	45.57 ± 14.6	**<0.001**
Female	39.46 ± 14.07	
**Age (years)**		
20–30	35.19 ± 14.29 ^a^	**<0.001**
31–40	44.39 ± 14.18 ^b^	
41–50	48.18 ± 12.21 ^bc^	
51–62	57.37 ± 4.34 ^c^	
**Sector**		
Primary health care center	42.34 ± 14.6	0.640
Hospital	43.02 ± 14.73	
**Type of facility**		
Primary health care center	42.34 ± 14.6	0.197
General hospital	42.64 ± 14.66	
Central hospital	43.48 ± 15.4	
Specialized hospital	56.6 ± 7.5	
**Receiving training on Notifiable Diseases Surveillance System (HESN+)**		
No	39.09 ± 14.05	**<0.001**
Yes	54.29 ± 9.77	
**Having any level of training or degree in epidemiology or disease surveillance**		
No	34.94 ± 13.22	**<0.001**
Yes	51.84 ± 10.41	
**Level of training or degree in epidemiology or disease surveillance**		
Certificate	52.39 ± 8.93 ^a^	**<0.001**
Diploma	49.09 ± 12.91 ^a^	
Bachelor’s degree	52.04 ± 9.64 ^a^	
Master’s degree	57 ± 9.6 ^a^	
Did not receive training	34.94 ± 13.22 ^b^	

Numerical data are presented as mean ± SD, Statistical significance at *p*-value < 0.05, Different lower-case letters indicate significant difference in pairwise comparison.

**Table 2:** Relation between demographic data and score for prevention and training skills on the NDSS.

Item	Prevention and Training Skills Score	*p*-Value
**Professional category**		
Physician	29.22 ± 7.04 ^a^	**<0.001**
Nurse	22.74 ± 8.72 ^b^	
Public health specialties	29.37 ± 6.63 ^a^	
Medical laboratory	23.16 ± 9.61 ^b^	
**Gender**		
Male	27.36 ± 8.28	**<0.001**
Female	23.6 ± 8.83	
**Age (years)**		
20–30	21.21 ± 8.81 ^a^	**<0.001**
31–40	26.46 ± 8.54 ^b^	
41–50	28.98 ± 6.82 ^b^	
51–62	33.75 ± 2.05 ^b^	
**Sector**		
Primary health care center	25.36 ± 9.06	0.604
Hospital	25.81 ± 8.52	
**Type of facility**		
Primary health care center	25.36 ± 9.06	0.353
General hospital	25.61 ± 8.51	
Central hospital	26.14 ± 8.79	
Specialized hospital	32.4 ± 5.03	
**Receiving training on Notifiable Diseases Surveillance System (HESN+)**		
No	23.69 ± 8.6	**<0.001**
Yes	31.74 ± 5.9	
**Having any level of training or degree in epidemiology or disease surveillance**		
No	21.32 ± 8.39	**<0.001**
Yes	30.64 ± 6.05	
**Level of training or degree in epidemiology or disease surveillance**		
Certificate	31.59 ± 5.17 ^a^	**<0.001**
Diploma	28.26 ± 7.17 ^a^	
Bachelor’s degree	30.62 ± 5.69 ^a^	
Master’s degree	33.93 ± 5.24 ^a^	
Did not receive training	21.32 ± 8.39 ^b^	

Values are presented as mean ± SD. Statistical significance was set at *p* < 0.05. Different lowercase superscript letters indicate statistically significant differences between groups based on pairwise comparisons.

**Table 3:** Relation between demographic data and total score for perceptions relating to the NDSS.

Item	Perception Score	*p*-Value
**Professional category**		
Physician	45.83 ± 6.26 ^ab^	**<0.001**
Nurse	43.79 ± 5.55 ^a^	
Public health specialties	46.74 ± 5.14 ^b^	
Medical laboratory	44.13 ± 5.74 ^a^	
**Gender**		
Male	45.48 ± 5.9	**0.045**
Female	44.36 ± 5.49	
**Age (years)**		
20–30	43.34 ± 5.95 ^a^	**<0.001**
31–40	44.95 ± 5.4 ^ab^	
41–50	46.5 ± 5.4 ^b^	
51–62	49.25 ± 5.39 ^b^	
**Sector**		
Primary health care center	44.97 ± 5.74	0.979
Hospital	44.96 ± 5.74	
**Type of facility**		
Primary health care center	44.97 ± 5.74	0.499
General hospital	44.92 ± 5.8	
Central hospital	44.59 ± 5.31	
Specialized hospital	48.8 ± 4.97	
**Receiving training on Notifiable Diseases Surveillance System (HESN+)**		
No	44.33 ± 5.76	**<0.001**
Yes	46.94 ± 5.2	
**Having any level of training or degree in epidemiology or disease surveillance**		
No	43.3 ± 5.61	**<0.001**
Yes	46.9 ± 5.27	
**Level of training or degree in epidemiology or disease surveillance**		
Certificate	46.62 ± 5.72 ^a^	**<0.001**
Diploma	45.39 ± 5.21 ^ab^	
Bachelor’s degree	48.04 ± 4.64 ^a^	
Master’s degree	47.4 ± 5.4 ^a^	
Did not receive training	43.3 ± 5.61 ^b^	

Values are presented as mean ± SD. Statistical significance was set at *p* < 0.05. Different lowercase superscript letters indicate statistically significant differences between groups based on pairwise comparisons.

**Table 4:** Linear regression analysis for factors associated with score for notification/case management skills on the NDSS.

Item	Univariate Analysis			Multivariable Analysis		
	Coefficient	95% CI	*p*-Value	Coefficient	95% CI	*p*-Value
**Professional category**						
Physician	Ref			Ref		
Nurse	−9.06	−12.88 to −5.25	**<0.001**	−6.9	−10.41 to −3.39	**<0.001**
Public health specialties	3.84	−0.24 to 7.92	0.065	−2.82	−6.63 to 0.99	0.147
Medical laboratory	−7.86	−12.14 to −3.59	**<0.001**	−6.01	−9.42 to −2.6	**0.001**
**Gender**						
Male	Ref			Ref		
Female	−6.11	−8.87 to −3.35	**<0.001**	1.02	−1.72 to 3.77	0.464
**Age (years)**						
20–30	Ref			Ref		
31–40	9.2	6.06 to 12.34	**<0.001**	3.49	0.77 to 6.22	**0.012**
41–50	12.99	9.68 to 16.3	**<0.001**	3.33	−0.09 to 6.74	0.056
51–62	22.19	12.49 to 31.88	**<0.001**	−1.02	−10.06 to 8.01	0.824
**Sector**						
Primary health care center	Ref			Ref		
Hospital	0.68	−2.18 to 3.54	0.64	−1.07	−3.2 to 1.06	0.323
**Receiving training on Notifiable Diseases Surveillance System (HESN+)**						
No	Ref			Ref		
Yes	15.2	12.24 to 18.15	**<0.001**	6.96	4.28 to 9.64	**<0.001**
**Having any level of training or degree in epidemiology or disease surveillance**						
No	Ref			Ref		
Yes	16.9	14.59 to 19.21	**<0.001**	7.42	4.15 to 10.68	**<0.001**
**Years of experience**	1.74	1.5 to 1.99	**<0.001**	0.97	0.65 to 1.3	**<0.001**

CI: Confidence interval, statistical significance at *p*-value < 0.05. Regression coefficients presented are unstandardized coefficients (B) with corresponding 95% confidence intervals.

**Table 5:** Linear regression analysis for factors associated with score for prevention and training skills on NDSS.

Item	Univariate Analysis			Multivariable Analysis		
	Coefficient	95% CI	*p*-Value	Coefficient	95% CI	*p*-Value
**Professional category**						
Physician	Ref			Ref		
Nurse	−6.49	−8.78 to −4.2	**<0.001**	−5.02	−7.24 to −2.81	**<0.001**
Public health specialties	0.15	−2.29 to 2.6	0.903	−4.03	−6.43 to −1.62	**0.001**
Medical laboratory	−6.06	−8.62 to −3.5	**<0.001**	−5.23	−7.38 to −3.08	**<0.001**
**Gender**						
Male	Ref			Ref		
Female	−3.76	−5.4 to −2.12	**<0.001**	−0.08	−1.81 to 1.65	0.928
**Age (years)**						
20–30	Ref			Ref		
31–40	5.25	3.37 to 7.12	**<0.001**	2.44	0.72 to 4.16	**0.006**
41–50	7.77	5.79 to 9.75	**<0.001**	3.06	0.91 to 5.21	**0.005**
51–62	12.54	6.75 to 18.33	**<0.001**	−0.32	−6.02 to 5.38	0.911
**Sector**						
Primary health care center	Ref			Ref		
Hospital	0.45	−1.26 to 2.16	0.604	−0.33	−1.68 to 1.01	0.627
**Receiving training on Notifiable Diseases Surveillance System (HESN+)**						
No	Ref			Ref		
Yes	8.05	6.25 to 9.86	**<0.001**	3.47	1.77 to 5.16	**<0.001**
**Having any level of training or degree in epidemiology or disease surveillance**						
No	Ref			Ref		
Yes	9.32	7.89 to 10.74	**<0.001**	5.17	3.11 to 7.23	**<0.001**
**Years of experience**	0.93	0.78 to 1.08	**<0.001**	0.4	0.19 to 0.6	**<0.001**

CI: Confidence interval, Statistical significance at *p*-value < 0.05. Regression coefficients presented are unstandardized coefficients (B) with corresponding 95% confidence intervals.

**Table 6:** Linear regression analysis for factors relating to perceptions regarding the NDSS.

Item	Univariate Analysis			Multivariable Analysis		
	Coefficient	95% CI	*p*-Value	Coefficient	95% CI	*p*-Value
**Professional category**						
Physician	Ref			Ref		
Nurse	−2.04	−3.61 to −0.47	**0.011**	0.19	−1.36 to 1.73	0.812
Public health specialties	0.9	−0.78 to 2.59	0.291	1.07	−0.59 to 2.74	0.206
Medical laboratory	−1.71	−3.47 to 0.06	0.058	0.59	−0.92 to 2.1	0.442
**Gender**						
Male	Ref			Ref		
Female	−1.13	−2.23 to −0.03	**0.045**	0.5	−0.69 to 1.68	0.408
**Age (years)**						
20–30	Ref			Ref		
31–40	1.61	0.32 to 2.9	**0.015**	−0.9	−2.09 to 0.28	0.135
41–50	3.15	1.79 to 4.52	**<0.001**	−0.72	−2.21 to 0.76	0.339
51–62	5.91	1.91 to 9.9	**0.004**	−0.49	−4.38 to 3.4	0.803
**Sector**						
Primary health care center	Ref			Ref		
Hospital	−0.02	−1.14 to 1.1	0.979	−0.26	−1.18 to 0.66	0.577
**Receiving training on Notifiable Diseases Surveillance System (HESN+)**						
No	Ref			Ref		
Yes	2.61	1.34 to 3.87	**<0.001**	−1.09	−2.28 to 0.1	0.072
**Having any level of training or degree in epidemiology or disease surveillance**						
No	Ref			Ref		
Yes	3.6	2.55 to 4.65	**<0.001**	−1.21	−2.66 to 0.24	0.101
**Years of experience**	0.47	0.37 to 0.58	**<0.001**	0.16	0.01 to 0.3	**0.034**
**Knowledge and skills**						
Notification and case management score	0.24	0.21 to 0.27	**<0.001**	0.21	0.14 to 0.27	**<0.001**
Prevention and training score	0.36	0.31 to 0.41	**<0.001**	0.09	−0.01 to 0.19	0.09

CI: Confidence interval, statistical significance at *p*-value < 0.05. Regression coefficients presented are unstandardized coefficients (B) with corresponding 95% confidence intervals.

## Data Availability

The data presented in this study are available on reasonable request from the corresponding author. However, access to the data is restricted due to its origin from hospital records, which involve confidentiality and institutional data protection policies. Therefore, the data can be shared upon justified request, subject to ethical approval and relevant permissions.

## Supplementary Materials

The following supporting information can be downloaded at: https://www.mdpi.com/article/10.3390/healthcare14131936/s1, File S1: Questionnaire.
