# Supplementary material for: Determinants of Healthcare Providers’ Notification, Prevention and Perception Scores Under the Notifiable Disease Surveillance System (NDSS) in Jazan, Saudi Arabia
Source: Healthcare (Basel). 2026 Jul 1;14(13):1936. doi: 10.3390/healthcare14131936 (PMC13362326; doi:10.3390/healthcare14131936)
Supplement: Supplementary file 1 [file healthcare-14-01936-s001.zip › healthcare-4293067-supplementary.pdf]

File S1: Questionnaire

Agree to participate in this questionnaire?

هل توافق على المشاركة في هذا الاستبيان ؟

- Yes
- No

Section A – Demographic Information:

In what professional category do you fall?

في أي فئة مهنية تقع؟

- Physician
- Nurse
- Public health specialties
- Medical laboratory

What is your gender?

ما هو جنسك؟

- Male
- Female

Your age in years:

كم عمرك بالسنوات:

- 20-30
- 31-40
- 41-50
- 51-62

In which sector are you employed?

في أي قطاع تعمل؟

- Primary health care center
- Hospital

In what type of facility do you work?

ماهو نوع المنشأة التي تعمل بها؟

- Primary health care center
- General Hospital
- Central Hospital
- Specialized Hospital

Did you ever receive training on Notifiable Diseases Surveillance System (HESN+)?

هل سبق لك ان تلقيت تدريب على نظام مراقبة الأمراض الواجب الإبلاغ عنها (حصن +)؟

- Yes
- No

Do you have any degree or formal training in epidemiology or disease surveillance?

هل لديك اي درجة علمية او تلقيت أي تدريب رسمي في علم الأوبئة أو مراقبة الأمراض؟

- Yes
- No

If you have any degree or received formal training in epidemiology or disease surveillance, what was the level of training or degree?

إذا كنت تمتلك أي درجة علمية أو تلقيت تدريبًا رسميًا في علم الأوبئة أو مراقبة الأمراض، فما هو مستوى التدريب أو الدرجة العلمية؟

- Certificate
- Diploma
- Bachelor's degree
- Master's degree
- Doctorate
- Not received training

How many years of experience do you have working in the Notifiable Diseases? (answer 0 if less than one year)

كم عدد سنوات الخبرة لديك في العمل في مجال الأمراض الواجب التبليغ عنها؟ (أجب بـ 0 إذا كانت أقل من سنة)

……………………………….

Section B – Practices related to the Notifiable Diseases Surveillance System:

Have you diagnosed, suspected or investigated any notifiable infectious disease in the last year?

هل قمت بالتشخيص أو الاشتباه أو التقصي بوجود أي مرض معدٍ خلال العام الماضي؟

- Yes
- No

If yes, which notifiable infectious diseases did you diagnosed, suspected or investigated?

إذا كانت الإجابة بنعم، ما هي الأمراض المعدية التي تم تشخيصها أو الاشتباه بها اوالتقصي عنها؟

…………………………………….

Did you notify the infectious disease?

هل قمت بالإبلاغ عن المرض المعدي؟

- Yes
- No

Did you know the chain of disease notification?

هل تعلم ما هو تسلسل التبليغ عن الأمراض؟

- Yes
- No

Who did you notify the diseases to?

لمن أبلغت عن الأمراض؟

…………………………………..

How long after diagnosed, suspected or investigated did you notify the disease?

بعد كم من الوقت من التشخيص أو الاشتباه او التقصي ابلغت عن المرض؟

- Immediately (first 4 hours)
- Not Immediately (within 72 hours)
- Weekly
- Monthly
- No diagnosed or suspected diseases
- The diseases were not notified

If you did not notify the diseases diagnosed, suspected or investigated, please state the reasons for not notifying:

إذا لم تقم بالإبلاغ عن الأمراض التي تم تشخيصها او الاشتباه بها، يرجى ذكر أسباب عدم الإبلاغ؟

- Limited access to required forms/documentation
- Lack of support
- Inadequate training
- Limited access to communication equipment
- All diseases notified
- No diagnosed or suspected diseases

Do you know how to notify through HESN plus?

هل تعرف كيفية الابلاغ من خلال حصن+؟

- Yes
- No

Do you have notification forms available in your facility/practice?

هل تتوفر نماذج الإبلاغ في منشأتك؟

- Yes
- No

Section C –Knowledge and Skills on the Notifiable Diseases Surveillance System (NDSS):

Below is a list of skills that you need and use as part of your participation in the NDSS. Please indicate what your current level of skill is to perform the task on a scale of 1-10 (with 1 being Low skills, needing more support or training) and 10 being (Very high skills, no support or training needed).

فيما يلي قائمة بالمهارات التي تحتاجها وتستخدمها كجزء من مشاركتك في نظام مراقبة الأمراض الواجب الإبلاغ عنها. يرجى الإشارة إلى مستوى مهارتك الحالي لأداء المهمة على مقياس من 1 إلى 10 (حيث 1 تعني مهارات منخفضة، تحتاج إلى مزيد من الدعم أو التدريب) و10 تعني (مهارات عالية جدًا، لا تحتاج إلى دعم أو تدريب)

	NOTIFICATION	MY SKILLS<br>Low Skills									Very High Skills
1.	**I know and understand the need for a Notifiable Disease Surveillance System**<br>**أنا أعلم وأفهم الحاجة إلى نظام مراقبة الأمراض التي يمكن الابلاغ عنها**	1	2	3	4	5	6	7	8	9	10
2.	**I know which diseases should be notified immediately on clinical suspicion**<br>**أعرف الأمراض التي يجب الإبلاغ عنها فورًا عند الاشتباه السريري**	1	2	3	4	5	6	7	8	9	10
3.	**I know which diseases should be notified within 72 hours of laboratory confirmation of diagnosis**<br>**أعرف الأمراض التي يجب الإبلاغ عنها خلال 72 ساعة من تأكيد التشخيص المخبري**	1	2	3	4	5	6	7	8	9	10
4.	**I know what process to follow in notifying a diseases**<br>**أعرف ما هي العملية التي يجب اتباعها للإبلاغ عن الأمراض**	1	2	3	4	5	6	7	8	9	10
5.	**Rate yourself on using HESN plus platform**<br>**قم بتقييم نفسك على استخدام منصة حصن بلس**	1	2	3	4	5	6	7	8	9	10
6.	**I am able to access the latest protocols and guidelines on notifiable diseases**<br>**أستطيع الوصول إلى أحدث البروتوكولات والمبادئ التوجيهية بشأن الأمراض التي يجب الإبلاغ عنها**	1	2	3	4	5	6	7	8	9	10
	PREVENTION AND TRAINING	**Low Skills**									**Very High Skills**
7.	**I am able to train other team members on the notification of diseases**<br>**أنا قادر على تدريب أعضاء الفريق الآخرين على الإبلاغ عن الأمراض**	1	2	3	4	5	6	7	8	9	10
8.	**I am knowledgeable on the prevention of notifiable diseases**<br>**لدي معرفة حول الوقاية من الأمراض التي يجب الإبلاغ عنها**	1	2	3	4	5	6	7	8	9	10
9.	**I am able to educate the patients on the prevention of notifiable diseases**<br>**أنا قادر على تثقيف المرضى حول الوقاية من الأمراض التي يجب الإبلاغ عنها**	1	2	3	4	5	6	7	8	9	10
10.	**I provide access for the patients to education material on the prevention of notifiable diseases in my facility**<br>**أقوم بتوفير إمكانية وصول المرضى إلى المواد التثقيفية حول الوقاية من الأمراض التي يجب الإبلاغ عنها في منشأتي**	1	2	3	4	5	6	7	8	9	10
11.	**I am able to train other team members on the prevention of communicable diseases**<br>**أنا قادر على تدريب أعضاء الفريق الآخرين على الوقاية من الأمراض المعدية**	1	2	3	4	5	6	7	8	9	10

Section D- Perceptions on the Notifiable Diseases Surveillance System (NDSS):

Listed below are statements on Attributes of the Notifiable Disease Surveillance. Using the provided scale state how strongly you agree or disagree with the statement.

فيما يلي قائمة بعبارات حول سمات مراقبة الأمراض الواجب الإبلاغ عنها. باستخدام المقياس المقدم، حدد مدى موافقتك أو عدم موافقتك على العبارة.

The form used to notify diseases is easy to understand

النموذج المستخدم للإبلاغ عن الأمراض سهل الفهم

- Strongly Disagree
- Disagree
- Neutral
- Agree
- Strongly Agree

The form used to notify diseases takes a long time to fill in

يستغرق ملء النموذج المستخدم للإبلاغ عن الأمراض وقتًا طويلاً

- Strongly Disagree
- Disagree
- Neutral
- Agree
- Strongly Agree

The notification process is easy to comply with

عملية الإبلاغ سهلة الامتثال بها

- Strongly Disagree
- Disagree
- Neutral
- Agree
- Strongly Agree

I am willing to participate in the Notifiable Disease Surveillance System

أنا على استعداد للمشاركة في نظام مراقبة الأمراض الواجب الإبلاغ عنها

- Strongly Disagree
- Disagree
- Neutral
- Agree
- Strongly Agree

Data obtained through the Notifiable Disease Surveillance System is used for outbreaks response

يتم استخدام البيانات التي تم الحصول عليها من خلال نظام مراقبة الأمراض الواجب الإبلاغ عنها للاستجابة لتفشي الأمراض

- Strongly Disagree
- Disagree
- Neutral
- Agree
- Strongly Agree

Outbreak response teams do respond timely to most outbreaks

تستجيب فرق الاستجابة للأوبئة في الوقت المناسب لمعظم حالات تفشي الأمراض

- Strongly Disagree
- Disagree
- Neutral
- Agree
- Strongly Agree

Data obtained through the Notifiable Disease Surveillance System is used for policy and guideline formulation

يتم استخدام البيانات التي تم الحصول عليها من خلال نظام مراقبة الأمراض الواجب الإبلاغ عنها لصياغة السياسات والمبادئ التوجيهية

- Strongly Disagree
- Disagree
- Neutral
- Agree
- Strongly Agree

Data obtained through the Notifiable Disease Surveillance System contribute to knowledge on the prevention and control of infectious diseases

تساهم البيانات التي تم الحصول عليها من خلال نظام مراقبة الأمراض الواجب الإبلاغ عنها في تعزيز المعرفة بشأن الوقاية من الأمراض المعدية ومكافحتها

- Strongly Disagree
- Disagree
- Neutral
- Agree
- Strongly Agree

The Notifiable Disease Surveillance System has been changed to meet changing circumstances and needs in the last three years

تم تغيير نظام مراقبة الأمراض الواجب الإبلاغ عنها لتلبية الظروف والاحتياجات المتغيرة في السنوات الثلاث الماضية

- Strongly Disagree
- Disagree
- Neutral
- Agree
- Strongly Agree

Lack of facility supervision do impact on compliance with the Disease Surveillance system

إن عدم وجود إشراف على المنشأة يؤثر على الامتثال لنظام مراقبة الأمراض

- Strongly Disagree
- Disagree
- Neutral
- Agree
- Strongly Agree

The department provided feedback to providers on notifiable diseases over the last year

قدم القسم ملاحظات لمقدمي الخدمات بشأن الأمراض التي يجب الإبلاغ عنها خلال العام الماضي

- Strongly Disagree
- Disagree
- Neutral
- Agree
- Strongly Agree

A high workload prevents me from notifying diseases

عبء العمل المرتفع يمنعني من الإبلاغ عن الأمراض

- Strongly Disagree
- Disagree
- Neutral
- Agree
- Strongly Agree

A lack of access to communication equipment prevents me from notifying diseases

إن عدم القدرة على الوصول إلى أدوات الاتصال يمنعني من الإبلاغ عن الأمراض

- Strongly Disagree
- Disagree
- Neutral
- Agree
- Strongly Agree
